# Effect of Cuttlebone on Healing of Indomethacin-Induced Acute Gastric Mucosal Lesions in Rats

**DOI:** 10.1155/2020/9592608

**Published:** 2020-10-07

**Authors:** Lifeng Qiu, Lingya Yao, Yanfei Fang, Lan Wang, Meng Xue, Zhenghua Lin, Shujie Chen, Jianmin Si

**Affiliations:** ^1^Department of General Practice, Sir Run Run Shaw Hospital, Zhejiang University, Hangzhou, Zhejiang 310000, China; ^2^Institute of Gastroenterology, Zhejiang University, Hangzhou, Zhejiang 310000, China; ^3^Department of Gastroenterology, Sir Run Run Shaw Hospital, Zhejiang University, Hangzhou, Zhejiang 310000, China; ^4^Department of Gastroenterology, Second Affiliated Hospital, Zhejiang University, Hangzhou, Zhejiang 310000, China

## Abstract

The continuing use of nonsteroidal anti-inflammatory drugs (NSAIDs) usually increases the side effects such as peptic ulcer and acute gastric lesions in the gastrointestinal tract. Cuttlebone (CB), isolated from Sepiella maindroni de Rochebrune, was reported to have antioxidant activities, but its role in the treatment of indomethacin-induced gastric lesions has not yet been confirmed. In this research, we investigate the protective effect of cuttlebone on indomethacin-related ulcers in rats and possible mechanisms. Here, gastric ulcers were induced by oral administration of indomethacin, and then the rats were treated with omeprazole (4 mg/kg) or different doses (750, 1500, and 3000 mg/kg of body weight) of cuttlebone. We evaluated lesion index, inflammation score, and a series of oxidant/antioxidant parameters. The data demonstrated that cuttlebone could protect against gastric ulcers induced by indomethacin in a dose-dependent manner (positive correlation). Also, these effects were associated with attenuating the expression of malonaldehyde (MDA) and increasing the levels of some protective ingredients like epidermal growth factor (EGF), prostaglandin E2 (PGE2), and superoxide dismutase (SOD). Thus, considering its ability to protect indomethacin-induced acute gastric mucosal lesions and the underlying mechanisms, CB might be a potential candidate for treating gastric damage caused by NSAIDs.

## 1. Introduction

Nonsteroidal anti-inflammatory drugs (NSAIDs) are proverbially used in rheumatic diseases, cardiovascular diseases, and the control of neoplasia development [1, 2]. However, gastrointestinal side effects come behind including duodenal ulcers or acute gastric mucosal lesions via inhibition of cyclooxygenase (COX) expression. COX‐1 was reported to be involved in mucus secretion, mucosal blood flow, and the maintenance of the gastric mucosa, while COX-2 serves as an inflammatory factor [3]. Thus, NSAIDs pose anti-inflammatory effects primarily via COX-2 inhibition, while the adverse effects occur simultaneously followed by COX-1 reduction such as local damage to the gastric mucosa. As one of the NSAIDs, indomethacin is prone to bring about serious side effects such as petechial bleeding, ulcerative lesions, and erosions in the mucosa of stomach despite the function of pain-relieving, anti-inflammatory, and antipyretic properties [4]. The development of the gastric mucosal layer lesions is chiefly regulated by the inhibition of prostaglandins and overproduction of free radicals [5]. According to previous reports, the inhibition of prostaglandin synthesis by indomethacin causes increased susceptibleness to mucosal lesions and gastroduodenal ulceration, as well as induction of oxidative stress and inflammation [6, 7]. Furthermore, an increase in oxidant levels and a decrease in antioxidant levels exhibit a correlation with the degree of damage to gastric mucosa [8].

Current treatment strategies for indomethacin-induced gastric mucosal lesions involve the use of proton pump inhibitors (PPIs), such as omeprazole, and antagonists of histamine receptor type 2 like ranitidine [9]. However, long-term continuous use of PPIs may increase the risks of fractures, thromboembolism, and even ulcer recurrence, which limit the use of pharmacological drugs [2, 10, 11]. Natural compounds with fewer adverse effects are more suitable than synthetic drugs for long‐term treatment in gastric lesions [12, 13]. Moreover, the cost of natural compound for gastric lesions is only one-sixth that of synthetic drugs [14]. Therefore, new effective and safe antiulcer therapeutic natural compounds are needed for the improvement of prevention of disease recurrence and the healing of gastric lesions [15].

Cuttlebone (CB), the internal structure isolated from Sepiella maindroni de Rochebrune, is traditional Chinese medicine used in the treatment of stomach ulcers, gastric hyperacidity, and a variety of bleeding [16]. It has previously been observed that CB displayed the wound healing activities in burnt lesions of rats by boosting the production of transforming growth factor-*β* (TGF-*β*) and vascular endothelial growth factor (VEGF) [17]. Moreover, previous research has established that CB had antioxidant property including scavenging hydroxyl radicals and chelating ferrous ions, which were also affiliated with the mechanism of indomethacin-induced acute gastric mucosal lesions. Antioxidant activities are attributed to the ability to engage hydrogen atoms from free radicals [18]. Also, cuttlebone could protect against hepatic injury caused by hepatotoxicants like carbon tetrachloride (CCl_4_) [19]. This effect was associated with the ability of CB to increase the activity of SOD. Clinical studies have showed that CB was effective to treat peptic ulcer, but the treatment mechanism is still needed to be studied. Also, the efficacy of CB in treating gastric mucosal lesions induced by NSAIDs has not yet been studied.

In the present study, we firstly established the model of indomethacin-induced gastric mucosal lesions in rats. Then, we observed the efficacy of CB with different doses in this model and measured the amount of EGF, PGE_2_, MDA, and SOD. We aim to test the feasibility of CB with its antiulcerative and therapeutic properties and to determine whether the antiulcer mechanism of CB is linked with EGF, PGE2, MDA, and SOD.

## 2. Materials and Methods

### 2.1. Preparation of CB

CB was first tested and processed through quality standards. Water was added into the material and boiled 3 hours for extraction. Then the extract was filtered using filter paper. The filtrates were combined and concentrated into the paste with a certain proportion. Furthermore, the paste was dried in a shaded area, sieved, and mixed in the form of particles. Finally, the extract was kept in a package until use.

### 2.2. Animals

A total of 60 male Sprague Dawley (SD) rats (150 ± 15 g) were supplied by Shanghai SLAC Laboratory Animal Co., Ltd (SCXK:2012-002). During the experiment, rats were provided with a specific pathogen-free state on a strict light cycle (lights on at 08:00 hours and off at 20:00 hours), temperature of 20–26, and humidity of 40–70%. After 24 h of food starvation, animals were treated with indomethacin (40 mg/kg dissolved in ddH_2_O) by gavage to establish the model of acute gastric mucosal injury as previously described [20]. All experimental protocols used in this experiment were reviewed and ratified by the Institutional Animal Care and Use Ethics Committee (IACUC) of the University of Zhejiang, China (ethical number: 2015-19). The care and use of all animals were in line with the Animal Research Reporting of In Vivo Experiments (ARRIVE) guidelines.

### 2.3. Experimental Procedure

We examined the antiulcerative effect of CB by adopting an indomethacin-induced mucosal lesion model in rats [20]. CB was administered by oral gavage at 750, 1500, and 3000 mg/kg dosages, respectively, in distilled water to groups of rats fasted for 24 h while 4 mg/kg omeprazole in phosphate buffer was given to the fourth group of rats fasted for 24 h simultaneously. After 30 minutes, 40 mg/kg indomethacin was given to each rat for all groups (750, 1500, and 3000 mg/kg cuttlebone groups, 4 mg/kg omeprazole group, and indomethacin control group) intragastrically in distilled water. An identical volume of distilled water was administered to the healthy group. Six hours after indomethacin ingestion, rats were narcotized with 4% chloral hydrate by intraperitoneal injection, and carprofen (5 mg/kg) was used by subcutaneous injection to relieve the pain of the rats simultaneously. Blood and tissue samples were collected.

### 2.4. Mucosal Lesion Analysis

The stomachs were cut off, spread along the greater curvature, and then fixed on a board for the purpose of the megascopic gastric lesion analysis. After repeatedly rinsing with ice saline, the tissue was fixed on a board, and the area of visible erosive lesions was gauged with a vernier caliper. Then, the lesion index was calculated in line with the method of Andrade et al. [21, 22]. Classification of ulcers was first described as ulcer area <1 mm^2^ (Level I), ulcer area = 1–3 mm^2^ (Level II), and ulcer area >3 mm^2^ (Level III), and then the ulcer indexes were calculated: ulcer index = 1 × (number of Level I ulcer) + 2 × (number of Level II ulcer) + 3 × (number of Level III ulcer).

### 2.5. Detection of Blood Specimen

To measure the levels of PGE2 and EGF (pg/ml) in blood serum, blood was obtained from arteria femoralis in rats and centrifuged (3000 rpm) for 10 min. After separation, the specimens were cryopreserved in vials at −20, followed by estimation using Rat-PGE2 and Rat-EGF enzyme-linked immunosorbent assay (ELISA) kits (purchased from Shanghai Baiwo Biotechnology Co., Ltd.).

### 2.6. Detection of Tissue Specimen

The concentration of PGE2, MDA, and SOD inhibition rate was measured by Rat-PGE2 ELISA kits (purchased from Shanghai Baiwo Biotechnology Co., Ltd.), thiobarbituric acid (TBA) method using MDA kit (purchased from Nanjing Jiancheng Institute of Biological Engineering), and water-soluble tetrazolium (WST-1) method using SOD kit (purchased from Nanjing Jiancheng Institute of Biological Engineering) and then divided by the protein concentration in tissue measured by bicinchoninic acid (BCA) protein quantification kit (purchased from Biyun Sky Institute of Biotechnology).

### 2.7. Histological Analysis

The stomach tissues were excised from SD rats and washed with ice saline. Later, the samples were fixed with 10% neutral buffered formalin, routinely processed with dehydration, and embedded in paraffin wax. Then the gastric tissue was fixed with 10% formalin, dehydrated in increasing concentrations of ethanol, paraffin-embedded, sectioned, stained with hematoxylin-eosin (HE), and scored according to Rauws classification standard [23]. Briefly, the inflammation score was assigned for each item to calculate as follows: (i) the infiltration density of inflammatory cells and monocytes in the mucosa lamina propria: 0–2 points; (ii) the infiltration density of the lamina propria neutrophils: 0–3 points; (iii) the density of neutrophils in the mucosa: 0–3 points; and (iv) the degree of superficial erosion: 0–2 points. The scoring criteria for each parameter were 0 = no, 1 = mild, 2 = medium, and 3 = heavy. A total of 0–10 changes should be used to measure inflammation degree.

### 2.8. Statistical Analysis

Statistical analysis was performed using one-way analysis of variance (ANOVA). All values were reported as the means ± standard deviation (SD). All statistical calculations were performed with Statistical Product and Service Solutions (SPSS) 13.0 software. *P* < 0.05 was considered to be statistically significant.

## 3. Results

### 3.1. CB Protects against Indomethacin-Induced Gastric Lesions

**Figure 1:**
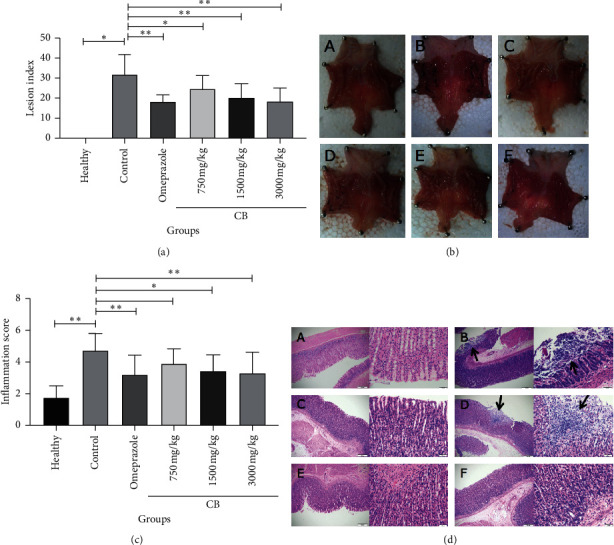
Effects of different doses of CB administration on indomethacin-induced lesions in rat stomachs. (a) The average lesion index. (b) Representative examples of stomach lesions: (A) healthy group, (B) control group, (C) omeprazole group, (D) low dose of CB group (750 mg/kg), (E) moderate dose of CB group (1500 mg/kg), and (F) high dose of CB group (3000 mg/kg). (c) The average inflammation score. (d) Representative examples of stomach stained with H&E at 100x and 400x magnification. Values are expressed as means ± SD. ^*∗*^*P* < 0.05 vs. control group and ^*∗∗*^*P* < 0.01 vs. control group.

To explore the curative function of CB on megascopic gastric lesions, we did macroscopic examination by ciphering lesion number and size on all rats in CB (750, 1500, and 3000 mg/kg) groups, omeprazole group, and control group. In all rats, the lesion foci were formed by mucosal defects like punctate- or linear-shaped and scattered to all of the stomach surfaces. In addition, hyperemia in the stomachs of rats in the control group was more distinct than that of rats in all other medicine groups. The average lesion index decreased with increasing CB dose (Figures 1(a) and 1(b)). We can see that the gastric mucosa of rats in the healthy group was smooth, the color was ruddy, and the mucosal fold was clear (Figure 1(b) A). As for rats in the control group, the lesions were textured of mucosal defects that were linear-shaped and dispersed to all stomach surfaces (Figure 1(b) B). When the rats were administered intragastrically with a low dose of CB (750 mg/kg), they showed punctate and short linear lesions (Figure 1(b) F). Rats in high and moderate doses of CB groups (3000 mg/kg and 1500 mg/kg, respectively) and omeprazole group all had slight punctate-shaped erosion with mild hyperemia (Figure 1(b) C, D, and E).

Next, the inflammation score of the gastric surface was computed to identify gastric inflammation for further demonstration of the curative effect of CB against indomethacin-induced gastric lesions. The average inflammation score decreased with increasing CB dose (Figures 1(c) and Figure 1(d)). At the dose of 3000 mg/kg, the anti-inflammation effects of CB were similar to those of omeprazole.

### 3.2. Effect of CB on EGF and PGE2 Induced by Indomethacin in Rat Serum

**Figure 2:**
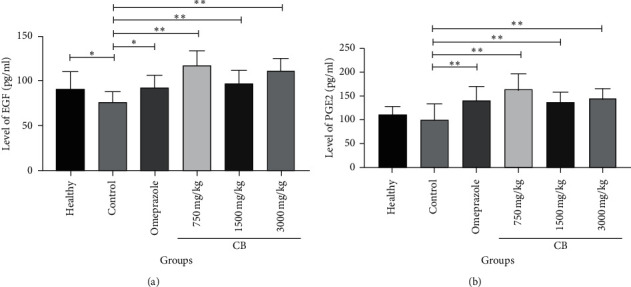
Effect of CB on EGF and PGE2 induced by indomethacin in rat serum. (a) The level of EGF in serum. (b) The level of PGE2 in serum. Values are expressed as means ± SD. ^*∗*^*P* < 0.05 vs. control group and ^*∗∗*^*P* < 0.01 vs. control group.

To determine whether CB would affect the secretion of EGF and PGE2, we measured the levels of EGF and PGE2 in rat serum. The concentrations of EGF were markedly reduced in the control group contrast to those in the healthy group (^*∗*^*P* < 0.05) (Figure 2(a)). In the omeprazole group, the concentrations of EGF were in elevated levels than those in the control group (^*∗*^*P* < 0.05) (Figure 2(a)). In the group with a low dose of CB (750 mg/kg), the concentration of EGF was obviously higher than the control group (^*∗∗*^*P* < 0.01) (Figure 2(a)). Similar results were seen in groups with moderate (1500 mg/kg) and high doses (3000 mg/kg) of CB (^*∗∗*^*P* < 0.01) (Figure 2(a)).

The concentrations of PGE2 in omeprazole and all groups of CB obviously increased when compared to those in the control group (^*∗∗*^*P* < 0.01) (Figure 2(b)).

### 3.3. Effect of CB on PGE2, MDA, and SOD Induced by Indomethacin in Gastric Mucosa

**Figure 3:**
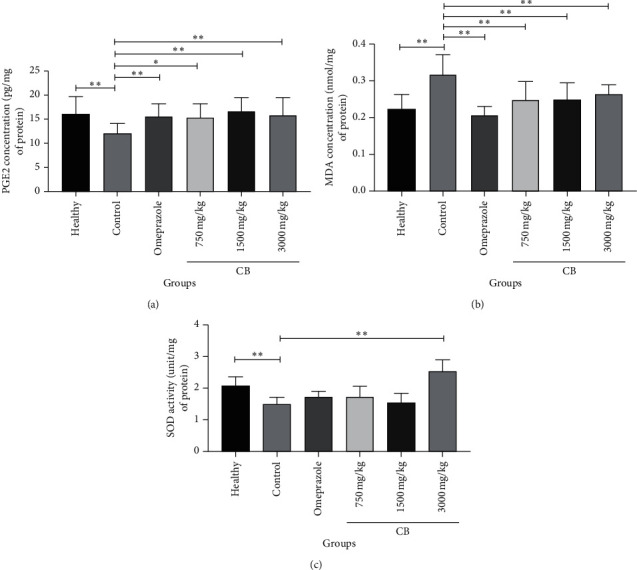
Effect of cuttlebone on PGE2, MDA, and SOD induced by indomethacin in the gastric mucosa. (a) The protein concentrations of PGE2 in the gastric mucosa. (b) The protein concentrations of MDA in the gastric mucosa. (c) The activities of SOD in the gastric mucosa. Values are expressed as means ± SD. ^*∗*^*P* < 0.05 vs. control group and ^*∗∗*^*P* < 0.01 vs. control group.

We measured the concentrations of PGE2, MDA, and SOD to clarify whether the antiulcer mechanism of CB was associated with them. The concentrations of PGE2 in gastric mucosa were markedly reduced in the control group compared with those in the healthy group (^*∗*^*P* < 0.05) (Figure 3(a)). The concentrations of PGE2 in omeprazole and high or moderate doses of CB groups obviously increased when compared to those in the control group (^*∗∗*^*P* < 0.01) (Figure 3(a)). In a low dose of the CB group, the concentrations of PGE2 were higher than those in the control group (^*∗*^*P* < 0.05) (Figure 3(a)).

The levels of MDA in healthy, omeprazole, and all doses of CB groups were similar and significantly lower than those in the control group (^*∗∗*^*P* < 0.01) (Figure 3(b)). Among all CB groups, the levels of MDA showed no obvious difference (Figure 3(b)).

The effect of CB on the effects of radical scavenging enzymes such as SOD in the gastric mucosa was also detected. Indomethacin treatment in the control group distinctly attenuated the activities of SOD in comparison with the healthy group (^*∗∗*^*P* < 0.01) (Figure 3(c)). The activities of SOD in the omeprazole group and all doses of CB groups all increased in comparison with those in the control group. But only the difference between a high dose of CB (3000 mg/kg) and the control group was statistically significant (^*∗∗*^*P* < 0.01) (Figure 3(c)).

## 4. Discussion

In China, about 3.6% of adults were reported to take NSAIDs periodically [24]. It has been studied that NSAIDs exert anti-inflammatory and calmative effects via COX-2 suppression, while inhibition of COX-1 by NSAIDs caused gastrointestinal toxicity [3]. Based on the fact that present treatments for indomethacin-induced gastric mucosal lesions are accompanied by adverse effects, including nephrotoxicity, hepatotoxicity, gynecomastia, and impotence [25], which limit their clinical application, new therapeutic strategies to prevent gastric lesions are urgently needed. Recently, there has been renewed interest in natural compounds because of fewer side effects and their broad spectrum of biological activity [26–29]. Here, we screened some natural compounds that have been reported to possess one or more bioactivities, including antibacterial, anti-inflammatory, and antioxidative activities for NSAID-induced gastric lesions treatment at clinically acceptable concentrations [16, 18, 30]. We found that CB, extracted from Sepiella maindroni de Rochebrune, showed a remarkable effect on protecting against indomethacin-induced acute gastric mucosal lesions. CB has been used in Chinese traditional medicine, and it was reported to exert some bioactivities, such as decreasing the wound size in scalpel-induced wound tissue in albino Wistar rats [31] and scavenging hydroxyl radicals and superoxide radicals [18]. In this study, as shown in Figure 1, we demonstrated that CB not only attenuated the inflammation response in the gastric mucosa but also decreased both the number and the size of gastric mucosal lesions. Furthermore, we showed a protective effect of CB against oxidative stress in vivo by using an indomethacin-induced gastric lesions rat model. The findings demonstrated that CB has a great potential value in the treatment of acute gastric mucosal gastric lesions caused by NSAIDs.

Indomethacin is one of the most common drugs to make an ulcerative model by producing gastric mucosal damage, the main mechanisms of which is overproduction of free radicals. By attaching close to ubiquinone and complex I of mitochondrial electron transport chain, indomethacin sums the formation of reactive oxygen species (ROS) through framing free radicals [32, 33]. Furthermore, studies have manifested that the generation of ROS can cause oxidative damage to the membranes (lipid peroxidation (LPO)), DNA, RNA, and protein molecules, accompanied by subsequent cell doom and injuries magnification [33, 34]. Besides, oxidative damage brings about protein oxidation as well as the generation of superoxide radicals, hydrogen peroxide (H_2_O_2_), and LPO, which will sabotage membrane lipids, DNA, cellular proteins, and organelles in a frank way [35].

PGE2, whose synthesis is catalyzed by COX-1, is responsible for gastric mucosal protection. Previous research studies had confirmed that the exogenous administration of PGE2 protected against indomethacin-caused gastric mucosal damage [36, 37]. EGF was reported to play an eventful role in healing gastric mucosa, including cell restoration, migration, and epidermization in recent years [38, 39]. Combined with our results, we speculate that CB can prevent gastric mucosal lesions via stimulating gastric protective factor EGF and relieving the inhibition of synthesis of PGE2.

The character of toxic oxygen radicals in the course of indomethacin-induced gastric damage has been confirmed [5]. According to previous research studies, the reduction of SOD expression aggravates gastric mucosal injuries and upregulates lipid peroxidation [40]. Also, a decline occurred in the SOD expression in gastric mucosal tissue after indomethacin administration [41]. Our results were consistent with these findings. The SOD expression in gastric mucosal tissue in a low or moderate dose of the CB group and omeprazole group did not show a significant difference compared with those in the control group, while the SOD activities increased largely after the rats were given a high dose of CB (3000 mg/kg). The results suggested that a high dose of CB (3000 mg/kg) could be better in activating antioxidant factors than omeprazole was. As the terminal product of lipid peroxidation, MDA is introduced to indicate its level [42, 43]. The level of MDA increased in indomethacin-induced gastric mucosal lesions. Being administered with omeprazole or CB could both significantly decrease the concentrations of MDA in gastric mucosa. Considering our results, we speculate that CB can protect against indomethacin-induced acute gastric mucosal lesions via the transcription factor nuclear factor erythroid-2-related factor 2 (Nrf2), which is a main regulator of cytoprotective responses to exogenous and endogenous stresses caused by electrophiles and ROS [44]. Nrf2 upregulates the expression of some cytoprotective proteins against ROS-induced oxidative stress and many antioxidant enzymes in the gastric mucosa [45]. The increased expression of these antioxidant enzymes was also observed in our research.

Although there are vital discoveries revealed by these studies, limitations still exist. First, we did not knock down the expression of PGE2, EGF, MDA, and SOD. Thus, saying that the antiulcer mechanism is directly interrelated with them may not be accurate. Second, to determine whether CB has severe side effects, we are supposed to treat the rats with CB periodically. Here, we only investigated the effects of CB on acute gastric mucosal lesions in a short time. The experiment should last for a time, and the results should include whether the rats show adverse effects.

## 5. Conclusions

Our study indicated that CB can protect against indomethacin-induced gastric injuries at different doses and the antiulcerative effect shows a positive correlation with different dosages. Furthermore, our results suggest a possibility of a correlation between the antiulcer mechanism and increased secretion of EGF and PGE2, prevention of lipid peroxidation, and activation of radical scavenging enzymes. Thus, CB, a natural compound extracted from a kind of cuttlefish, may be a potential therapeutic candidate for preventing and treating indomethacin-induced acute gastric mucosal lesions.

## Data Availability

The data used to support the findings of this study can be accessed through https://doi.org/10.7910/DVN/Q3UKI2, Harvard Dataverse.
